# Interaction of Phytophagous Insects with *Salmonella enterica* on Plants and Enhanced Persistence of the Pathogen with *Macrosteles quadrilineatus* Infestation or *Frankliniella occidentalis* Feeding

**DOI:** 10.1371/journal.pone.0079404

**Published:** 2013-10-24

**Authors:** José Pablo Soto-Arias, Russell Groves, Jeri D. Barak

**Affiliations:** 1 Department of Plant Pathology, University of Wisconsin-Madison, Madison, Wisconsin, United States of America; 2 Department of Entomology, University of Wisconsin-Madison, Madison, Wisconsin, United States of America; University of Malaya, Malaysia

## Abstract

Recently, most foodborne illness outbreaks of salmonellosis have been caused by consumption of contaminated fresh produce. Yet, the mechanisms that allow the human pathogen *Salmonella enterica* to contaminate and grow in plant environments remain poorly described. We examined the effect of feeding by phytophagous insects on survival of *S. enterica* on lettuce. Larger *S. enterica* populations were found on leaves infested with *Macrosteles quadrilineatus*. In contrast, pathogen populations among plants exposed to *Frankliniella occidentalis* or *Myzus persicae* were similar to those without insects. However, on plants infested with *F. occidentalis*, areas of the infested leaf with feeding damage sustained higher *S. enterica* populations than areas without damage. The spatial distribution of *S. enterica* cells on leaves infested with *F. occidentalis* may be altered resulting in higher populations in feeding lesions or survival may be different across a leaf dependent on local damage. Results suggest the possibility of some specificity with select insects and the persistence of *S. enterica*. Additionally, we demonstrated the potential for phytophagous insects to become contaminated with *S. enterica* from contaminated plant material. *S. enterica* was detected in approximately 50% of all *M. quadrilineatus*, *F. occidentalis*, and *M. persicae* after 24 h exposure to contaminated leaves. Particularly, 17% of *F. occidentalis*, the smallest of the insects tested, harbored more than 10^2^ CFU/F*. occidentalis*. Our results show that phytophagous insects may influence the population dynamics of *S. enterica* in agricultural crops. This study provides evidence of a human bacterial pathogen interacting with phytophagous insect during plant infestation.

## Introduction

The frequency and severity of produce-related foodborne illness outbreaks have increased in the last few decades [1,2]. Although consumption of fruits and vegetables has risen in recent years, these well-publicized foodborne outbreaks trigger consumer concerns about the safety of fresh produce, and impose a negative impact on the agricultural sector. In the US, *Salmonella enterica* is the number one cause of bacterial food-borne illness, and the incidence of infection has not declined over the past 15 years, and instead, has increased slightly since mid-2000s [3,4]. Recently, fresh produce has been linked to more salmonellosis outbreaks than any animal product; and now, plants are considered an important part of the life cycle of enteric human pathogens and as vectors to humans [5].

 Human pathogens experience harsh conditions on the leaves of field-grown plants, and survival may depend on tri-trophic interactions. Net growth of *S. enterica* on leaves is rare and populations tend to decline steadily overtime [6-8]. This suggests that multiplication factors are required to induce growth of bacterial populations or sustain infectious populations for extended periods, in what is normally described as a non-host environment. Liberation of plant nutrients by physical damage or plant pathogen infection has been shown to influence the survival of human pathogens [9-12]. The role of additional biological multipliers, such as phytophagous insects remains unexplored.

 Bacteria have evolved to exploit insects as hosts and/or vectors. Several studies have found an intimate relationship between insects and members of the Enterobacteriaceae [13], the family to which *Salmonella* belongs. In fact, numerous insects, such as flies, beetles and cockroaches, are associated with human habitations and livestock facilities, and have been linked with the spread of *S. enterica* [14]. In all these studies, mechanical transfer of the bacterium on body surfaces after contact with contaminated materials has been suggested as the likely mechanism for movement [14-16]. Several phytophagous insects are considered as widespread pests of agricultural crops many of which are known to be competent vectors of plant pathogens, including members of the Enterobacteriaceae [13,17]. Insect feeding on plants raises the possibility of a biological interaction, in addition to simple physical contamination, between *S. enterica* and phytophagous insects [18].

 Specifically, insect-feeding activity may influence foodborne pathogen populations on leaves. Feeding sites could represent a preferential niche that would allow bacterial multiplication due to access to nutrients liberated from surrounding damaged plant cells or protein/carbon-rich substances excreted during or after feeding by insects [18,19]. The effect of insect feeding on growth of human enteric bacteria on plant surfaces has been documented, however, only with *Escherichia coli*. Wasala and collaborators [19] reported that regurgitation spots of house flies (*Musca domestica*) represent a nutrient source that allows *E. coli* O157:H7 to multiply on spinach leaves. Additionally, Erickson et al. [18] observed higher *E. coli* O157:H7 populations on lettuce leaves that were inoculated soon after being fed upon by cabbage loopers (*Trichoplusia ni*). The effect of feeding by phytophagous insects on contaminated plants has not been studied, and the potential for insect activity to act as a ‘biomultiplier’ of *S. enterica* on agricultural crops remains unknown. 

 In this study, we investigated the effect of feeding by phytophagous insects on survival of *S. enterica* in the phyllosphere. We chose lettuce as our model phyllosphere because it is a common host to the three phytophagous insects we chose as representative cell-content and phloem-sap feeders [20-22] and leafy greens are responsible for 23% of the foodborne illness outbreaks associated with contaminated produce [23]. Because the type of mouthparts will also determine the type of damage caused by the insect, and therefore, potentially influence bacterial populations, we examined the interaction of *S. enterica* with two types of insect feeding. Thrips are cell-content feeders that induce a condition described as ‘silvering’ on leaves, resulting from feeding damage using rasping-sucking mouthparts that damage surface epithelial cells. Hemipteran insects, such as aphids and leafhoppers, are phloem-sap feeders that ingest plant fluids without severe cellular damage to mesophyll cells. We found larger *S. enterica* populations on leaves co-infested with *Macrosteles quadrilineatus*. On plants infested with *Frankliniella occidentalis*, areas of silvering harbored higher *S. enterica* populations than areas without lesions. We also observed that insect feeding type did not influence insect contamination rates. However, *S. enterica* populations on individual insects varied by 2 logs. 

## Materials and Methods

### Bacterial strains, media, and culture conditions

Six *S. enterica* serovars Cubana strain 98A9878 [24], Enteritidis strain 99A-23 (California Health Department [CHD], July 2005 tomato outbreak), Newport strain 96E01152C-TX [25], Poona strain 00A3563 (CHD, cantaloupe outbreak), Schwarzengrund strain 96E01152C [21], Baildon strain 05x-02123 [26] and Mbandaka strain 99A1670 (CHD, alfalfa seed isolate) were used in this study. These strains were selected because they were responsible for salmonellosis outbreaks associated with contaminated fresh produce. Bacterial cultures were grown overnight on Luria-Bertani (Difco/Becton Dickinson, Franklin Lakes, NJ) agar containing kanamycin (50 mg/liter) at 37°C. *S. enterica* strains were suspended from plates in sterile water to an optical density of 0.2 at 600 nm, which approximates 10^8^ CFU/ml. *S. enterica* strains were always inoculated as a six-strain cocktail at 1:1:1:1:1:1 ratio per strain. A *S. enterica* strain cocktail was used to mitigate possible strain differences in the plant-microbe-insect interaction. Xylose Lysine Desoxycholate (XLD) agar (Difco), a *Salmonella* semi-selective growth medium in which all chosen strains produce black colonies, was used to determine *S. enterica* populations from both leaf and insect samples. To verify that the black colonies recovered with XLD were the inoculated strains, each strain was transformed with pKT-Kan that confers kanamycin resistance and constitutive green fluorescent protein expression [27] without affecting the survival and growth of *S. enterica* on roots [28]. 

### Insect rearing

A *Frankliniella occidentalis* Pergande (Thysanoptera: Thripidae) colony was maintained on green bean pods (*Phaseolus vulgaris* L.) on the campus of the University of Wisconsin, Madison, Wisconsin. *F. occidentalis* colonies were maintained in plastic deli cups under ambient temperature and a 16:8 (L:D) photoperiod as previously described [29]. A colony of *Macrosteles quadrilineatus* Forbes (Hemiptera: Cicadellidae) was maintained on oat (*Avena sativa* L.) seedlings in a controlled environment with a 16:8 (L:D) photoperiod (24°C light; 19°C dark) [30]. A colony of *Myzus persicae* Sulzer (Hemiptera: Aphididae) was kindly provided by Dawn M. Smith (Cornell University), and established and maintained on Chinese cabbage (*Brassica rapa*) under similar controlled conditions as the *M. quadrilineatus* colony on the campus of the University of Wisconsin, Madison, Wisconsin.

### Lettuce plant inoculation

Lettuce plants (*Lactuca sativa* cultivar butterhead), were cultivated in a growth chamber without insecticide treatments. Three-week-old plants were dip-inoculated with either sterile water, as a control, or a *S. enterica* cocktail suspension for 1 min. Plants were allowed to dry under a laminar flow hood and then kept in transparent plastic boxes at 25°C with covers on top to maintain high humidity for 24 h. *S. enterica* inoculum was verified by serial dilution and plated on LB-kan agar before and after plant dip-inoculation to ensure that the bacterial concentration was constant throughout the inoculation process. 

### Feeding experiments

Lettuce plants were dip-inoculated as described above. Twenty-four hours post inoculation, adult *F. occidentalis* were transferred to half of the lettuce plants at a density of 25 individuals per plant. *F. occidentalis*-infested and *F. occidentalis*-free plants were confined using a cage consisting of a 15 cm-diameter plexiglass tube covered with *F. occidentalis*-proof mesh that surrounded an individual plant and eliminated insect escape and plant exposure to unintended infestation*. S. enterica* leaf populations were enumerated prior to insect infestation, referred to as 0 day post infestation (dpi), and periodically at 4, 9, and 13 dpi. At each sampling time, two 5 mm-diameter leaf discs were excised from each plant. All samples were homogenized in 500 μl of sterile water, and dilution plated on XLD-kan agar, and incubated at 37°C overnight for bacterial-population enumeration. From *F. occidentalis*-infested plants, leaf discs were sampled for *S. enterica* from leaf areas with visible *F. occidentalis* feeding damage (silvering). Each treatment consisted of 8 plants individually potted, which were randomly arranged and exposed to the same conditions. All experiments were repeated at least three times. 

The same protocol was followed, as described above, in a separate set of replicated experiments (silvering +/-), except adult *F. occidentalis* were added to all plants. Leaf discs were sampled for *S. enterica* from areas with and without *F. occidentalis* feeding damage within the same leaf. Eight, individually potted plants were used for each experiment and the experiment was repeated three times.

In a separate set of replicated experiments, adult *M. quadrilineatus* and *M. persicae* were transferred to half of the *Salmonella-*inoculated plants and confined in individual clip-cages at a density of 4 *M. quadrilineatus* and 5 *M. persicae* per cage and 3 cages per plant. Empty cages were placed on insect-free plants. *S. enterica* populations were enumerated prior to insect introduction (0 dpi) and periodically at 2, 4 and 9 dpi as described above. Each treatment consisted of 8 plants planted in individual pots, which were randomly arranged and exposed to the same conditions. All experiments were repeated four times.

### Microscopy

A subset of leaves from *S. enterica* inoculated plants were collected and examined microscopically with an Olympus BX-60 epifluorescence microscope (Opelco, Dulles, VA). In order to identify preferred colonization sites on leaves from plants exposed and non-exposed to insects, leaf tissue was mounted on microscopic slides and examined for green fluorescence from bacteria as previously described [31].

### Insect contamination

Lettuce plants were dip-inoculated as described above. Ten leaves were carefully removed from control and inoculated plants 24 hours after inoculation with a sterile razor blade. Individual leaves were placed inside a sterile petri dish. Non-contaminated insects were collected from respective colonies, and placed in the bottom of each dish containing either the mock or a *S. enterica*-inoculated leaf at different densities due to different insect sizes (*F. occidentalis*= 10, *M. quadrilineatus*= 5, *M. persicae*= 7 per dish). Petri dishes were sealed with a strip of parafilm to prevent insect escape. Live insects were collected in sterile microcentrifuge tubes after a 24 h exposure to *S. enterica*-inoculated leaves, placed at -80°C for 30 min to kill them without affecting potential surface contamination, and enriched in LB overnight at 37°C. Insects in enrichment broth were homogenized and a sterile loop was used to streak the enriched sample onto XLD-kan to verify the presence of *S. enterica* in or on insect bodies. Appearance of black colonies 24 h post-streaking were scored as *S. enterica* positive. A subset of random *S. enterica* presumptive positive, black colonies was confirmed by PCR using primers that target the *inv*A gene of *Salmonella* as previously described [32]. Additionally, leaf samples were collected and plated on XLD-kan agar before insects were added and after they were collected, to verify that inoculated leaves were contaminated with *S. enterica* and control leaves were not. Each treatment consisted of 10 petri dishes containing insects and the experiments were repeated four (*F. occidentalis*) or five (*M. persicae* and *M. quadrilineatus*) times, resulting in a minimum of 200 insects per treatment.

*S. enterica* population size per insect was determined following feeding on contaminated produce. Specifically, green bean pods were surface-sterilized by dipping in 10% bleach solution for 10 min, and placed in individual 50 ml conical tubes containing either 6 ml of sterile water or *S. enterica* suspension (prepared as described above). Conical tubes were placed horizontal in a shaking incubator at 37°C at 200 rpm overnight. Green beans were removed from the liquid and allowed to dry under a laminar flow hood and then placed in new sterile conical tubes. *F. occidentalis* were collected from the corresponding colony, and added to the conical tubes containing either the mock or the *S. enterica*-inoculated beans at a density of 15 *F. occidentalis* per tube. *F. occidentalis*-proof mesh was fixed to the tube cap to prevent insect escape or death from suffocation. Furthermore, green beans samples were collected, serially diluted, and plated on XLD-kan before insects were added and after they were collected, to verify that inoculated green beans were contaminated with *S. enterica* and control green beans were not. Live insects were collected in sterile microcentrifuge tubes after a 24 h contamination period, and placed at -80°C for 30 min to kill them without affecting potential surface contamination. Then, insects were homogenized in sterile water and *S. enterica* populations per insect were enumerated directly on XLD-kan. A subset of random *S. enterica* presumptive positive, black colonies was confirmed by PCR as described above. Each treatment consisted of 6 conical tubes and the experiment was repeated three times, resulting in a minimum of 100 insects per treatment.

### Statistical analysis

To determine whether the average population or incidence of *S. enterica* differed between treatments or over time, analysis of covariance (ANCOVA) was used to test the potential effects of insect feeding on *S. enterica* populations on leaves, with treatment and time (dpi) as covariates. Bacterial counts were log transformed prior to analysis and repetitions of the experiment were considered as block factors. In this manuscript, the intercept parameter is described as the starting *S. enterica* population, and the slope parameter is described as a measure of *S. enterica* population persistence. In the special instance where both silvered and non-silvered leaf tissue was sampled, leaf samples from the same plants were randomly assigned to one of the two treatments prior to insect addition. Therefore, in the analysis of the silvering assay, the intercept was estimated in the same way, but the model was modified to disallow variation between treatments for the y-intercept. For the insect contamination experiments, a two-tailed *Z* test (critical value ± 1.96) was used to test if the percentage of contaminated insects was statistically higher than 50% of the total population of insects tested. All statistical analysis were performed using R software [33].

## Results

### Extended survival of *S. enterica* on lettuce leaves in areas damaged by cell-content feeders

**Figure 1:**
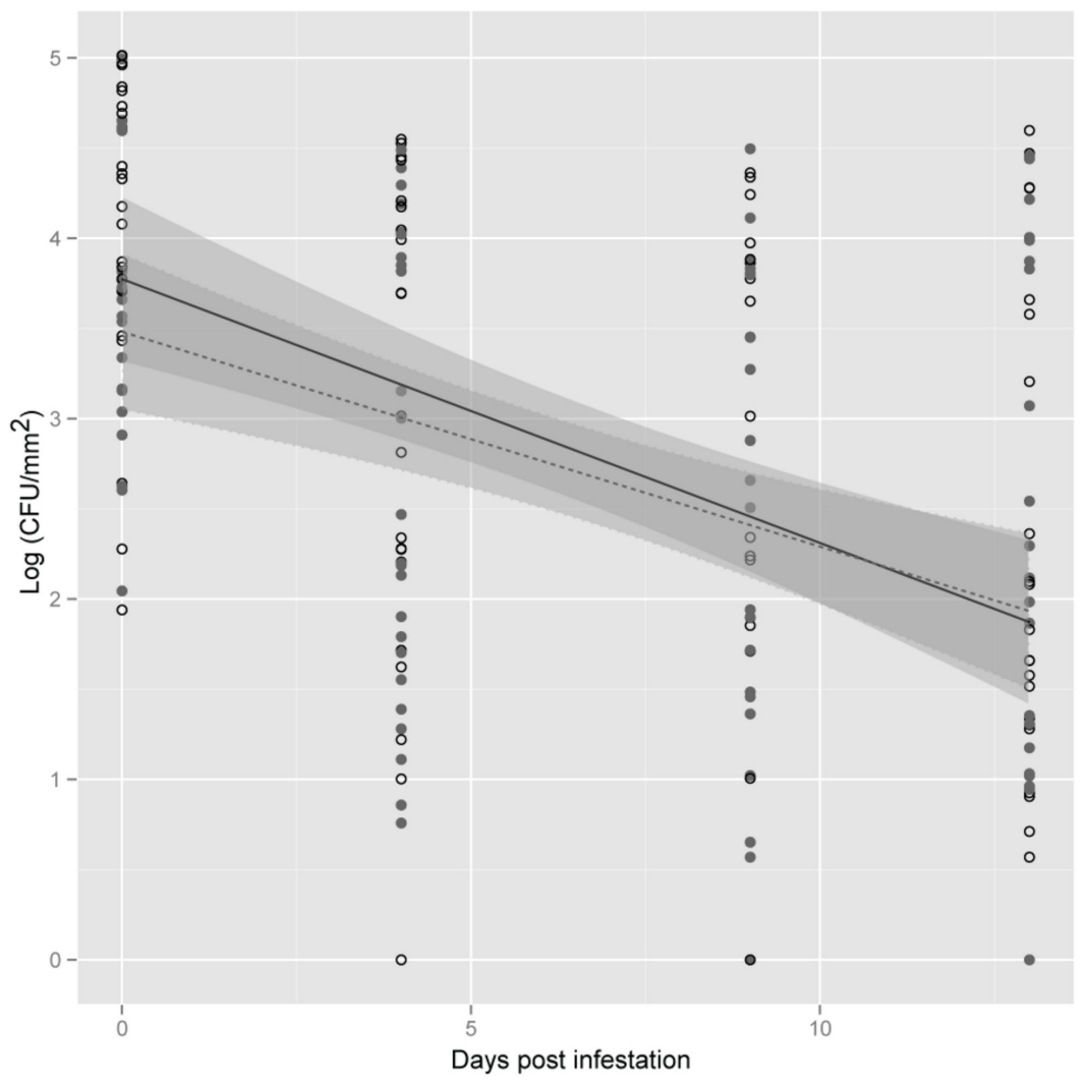
*Salmonella enterica* population dynamics on plants exposed to *Frankliniella occidentalis*. Lettuce plants were exposed (open circles) or non-exposed (close circles) to *F. occidentalis*. Shown is the mean log population of *S. enterica* on lettuce leaf samples (CFU/mm^2^) at 0, 4, 9 and 13 days post infestation. The data represent the means of three independent experiments. Lines (black, exposed; dotted, non-exposed) correspond to a linear regression model, and shaded areas to their associated 95% confidence interval.

Lettuce plants inoculated with *S. enterica* were exposed to *F. occidentalis* to investigate if insect infestation influenced *S. enterica* populations. Although plants were inoculated at a high concentration (10^8^ CFU/ml), bacterial populations that colonized leaves at the beginning of the insect infestation interval (0 dpi), averaged 10^4^ CFU/mm^2^. *S. enterica* was not recovered from uninoculated control plants (data not shown). In the case of inoculated plants, *S. enterica* populations declined over time. Surprisingly, the final bacterial concentrations did not reach zero, even 14 days after inoculation. In addition to differences in bacterial populations over the sampling interval (dpi *P*<0.05); the slopes varied among experimental replications (exp:dpi *P*<0.05; Figure 1). However, no significant differences were observed in *S. enterica* populations (*P*>0.05) or population decline over time among plants exposed or non-exposed to *F. occidentalis*, evident in the interaction trt:dpi (*P*>0.05, Figure 1). *F. occidentalis* were freely released onto whole plants instead of being confined on individual leaves, or portions of leaves, allowing them to feed in an unrestricted manner over the entire plant. In turn, samples from the same plant were collected from different leaves, because of a lack of sufficient *F. occidentalis* feeding sites (silvering) on the same leaf potentially increasing the variability among samples. 

**Figure 2:**
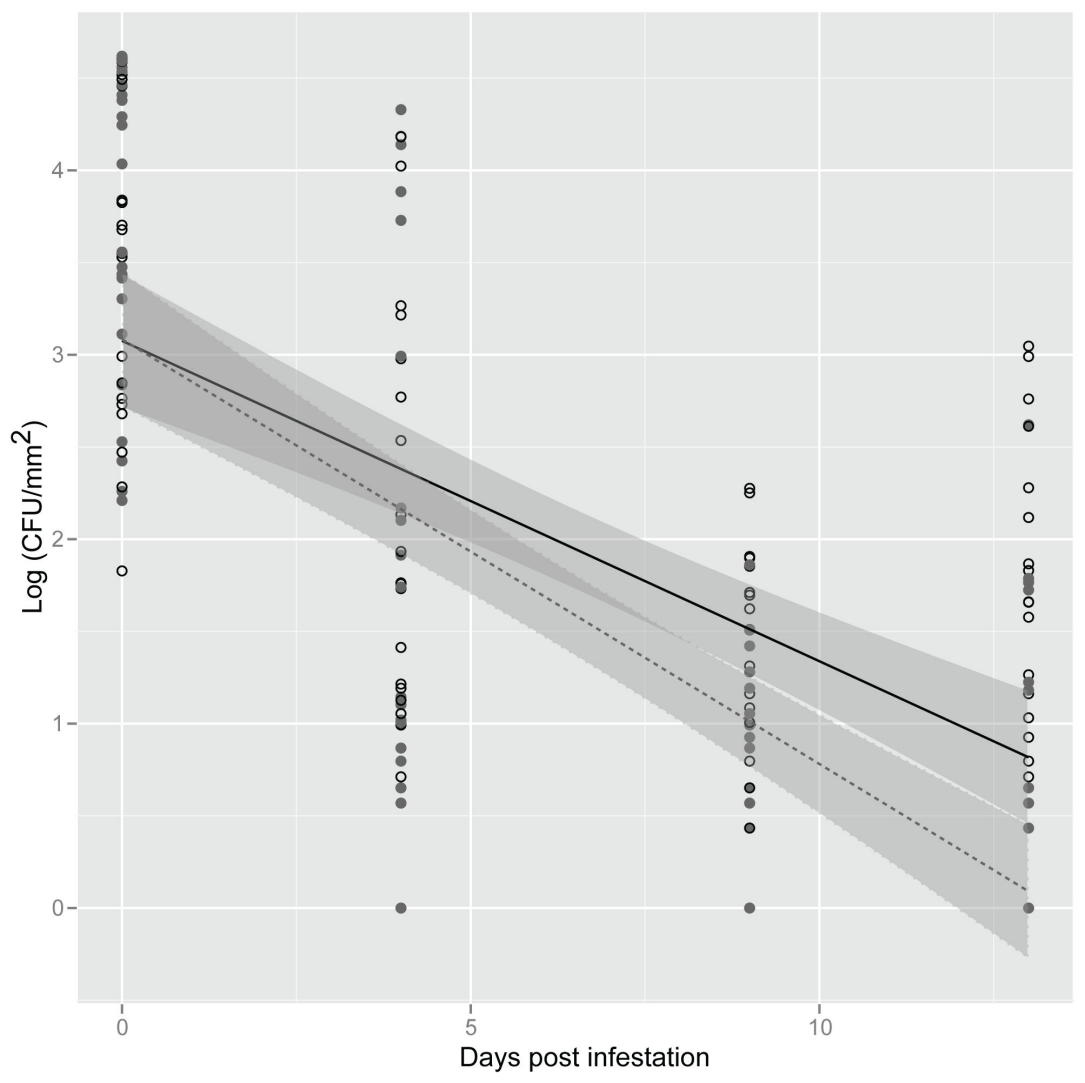
Extended survival of *Salmonella enterica* on lettuce leaves in areas damaged by *Frankliniella occidentalis*. Open circles represent areas with feeding damage and close circles areas without feeding damage caused by *F. occidentalis*. Shown is the mean log population of *S. enterica* on lettuce leaf samples (CFU/mm^2^) at 0, 4, 9 and 13 days post infestation. The data represent the means of three independent experiments. Lines (black, exposed; dotted, non-exposed) correspond to a linear regression model, and shaded areas to their associated 95% confidence interval.

 However, using fluorescent microscopy, we consistently observed the presence of *gfp*-tagged *S. enterica* cells accumulating in areas that were fed upon by *F. occidentalis* (data not shown); therefore, the effect of *F. occidentalis* feeding damage was further investigated. Survival of *S. enterica* in feeding areas with obvious silvering was compared with areas without visible feeding damage in the same plant. Similar to our earlier findings, bacterial populations declined over the sampling interval (dpi *P*<0.05). But in contrast to the experiments with or without *F. occidentalis*, bacterial populations were similar among experimental replications (*P*>0.05) in these experiments. Overall, *S. enterica* population decline was delayed in areas with silvering (trt:dpi interaction, *P*<0.05), and sustained significantly higher bacterial populations even at 10 and 13 dpi when compared to undamaged areas (Figure 2). Consistently, *S. enterica* was not recovered from uninoculated control plants.

### Enhanced survival of *S. enterica* on lettuce plants in the presence of certain phloem-sap feeding insects

**Figure 3:**
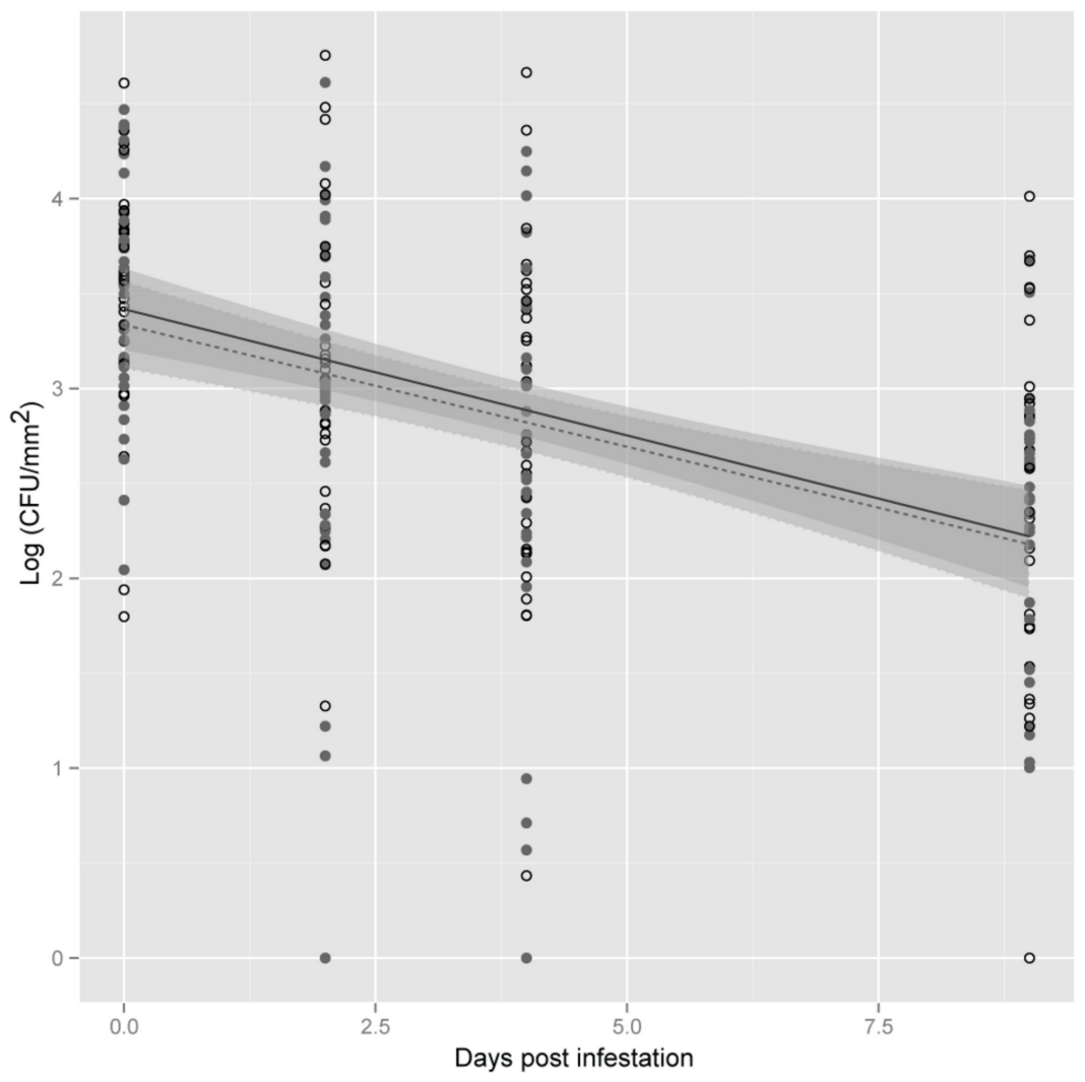
*Salmonella enterica* population dynamics on plants exposed to *Myzus persicae*. Lettuce plants were exposed (open circles) or non-exposed (close circles) to *M. persicae*. Shown is the mean log population of *S. enterica* on lettuce leaf samples (CFU/mm^2^) at 0, 2, 4 and 9 days post infestation. The data represent the means of four independent experiments. Lines (black, exposed; dotted, non-exposed) correspond to a linear regression model, and shaded areas to their associated 95% confidence interval.

**Figure 4:**
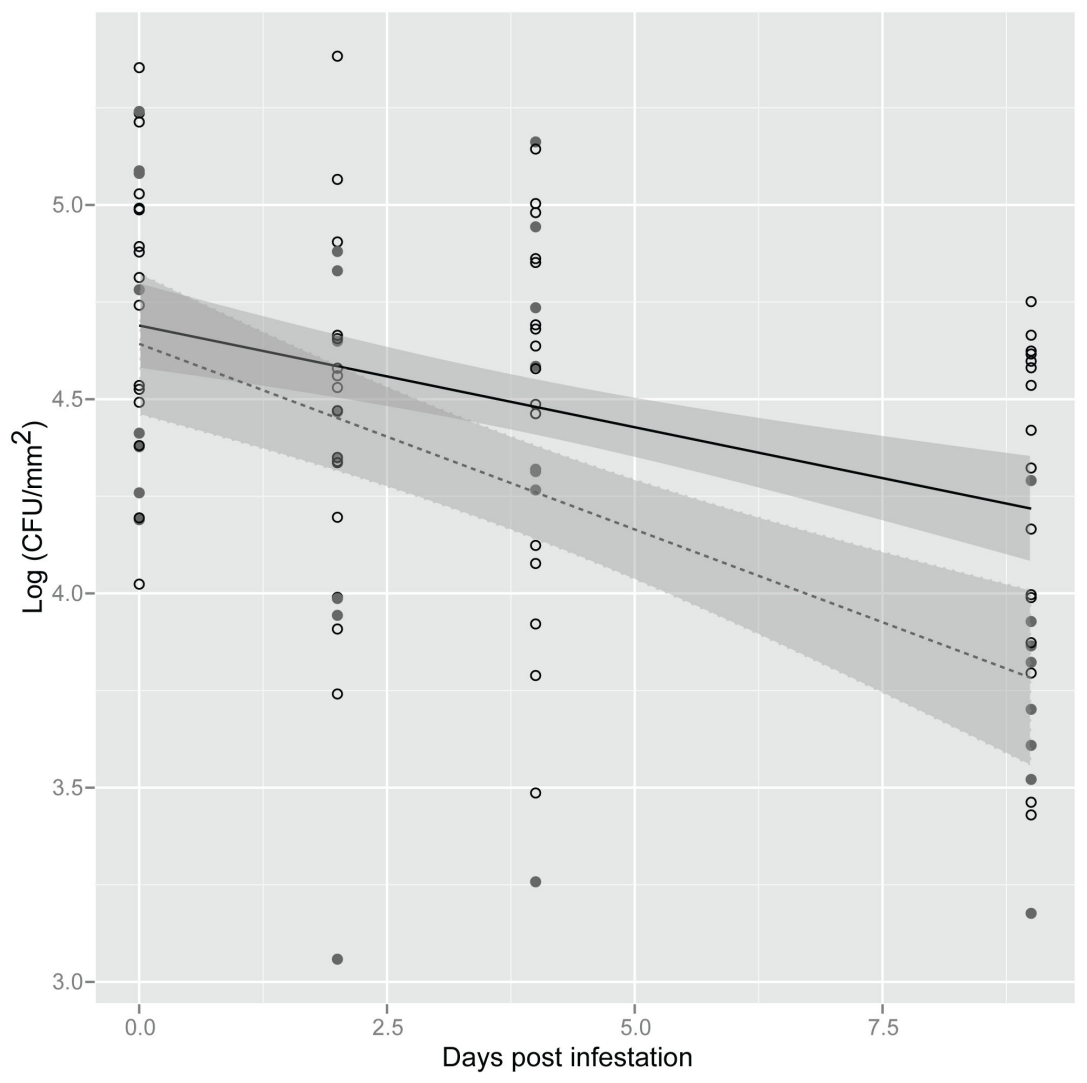
Increased survival of *Salmonella enterica* on plants exposed to *Macrosteles quadrilineatus*. Lettuce plants were exposed (open circles) or non-exposed (close circles) to *M. quadrilineatus*. Shown is the mean log population of *S. enterica* on lettuce leaf samples (CFU/mm^2^) at 0, 2, 4 and 9 days post infestation. The data represent the means of four independent experiments. Lines (black, exposed; dotted, non-exposed) correspond to a linear regression model, and shaded areas to their associated 95% confidence interval.

To determine if *S. enterica* survival on lettuce was specific or unique to *F. occidentalis* feeding, *S. enterica* population survival was examined in plants exposed to phloem-feeding insects. In experiments where *S. enterica*-inoculated plants were exposed to *M. persicae*, significant variation in bacterial populations was observed along sampling days (dpi, *P*<0.05; Figure 3). However, there were no significant differences in *S. enterica* populations between treatments (*P*>0.05) or in population decline over time among plants exposed or non-exposed to *M. persicae* (trt:dpi, *P*>0.05). In contrast, exposure of plants to *M. quadrilineatus* enhanced *S. enterica* survival compared to plants that were not exposed to insects (*P*<0.05; Figure 4). Likewise, the rate of decline of *S. enterica* was significantly attenuated in the presence of *M. quadrilineatus* (trt:dpi, *P*<0.05), resulting in approximately half a log higher bacterial populations at 13 dpi in *M. quadrilineatus*-exposed plants. 

### Phytophagous insects become contaminated and harbor elevated *S. enterica* populations from contaminated produce

The potential for phytophagous insects to become contaminated with *S. enterica* from contaminated plant material was observed by detection of the bacteria in approximately 50% of all insects tested. A total of 241 *F. occidentalis*, 229 *M. quadrilineatus*, and 289 *M. persicae* were exposed to contaminated lettuce leaves for 24 h, and subsequently tested for the presence of *S. enterica*. Dead insects were not collected to assure that sampled insects were in contact with contaminated plant tissue. *S. enterica* was not isolated from untreated control treatments. From the exposed insects, 52% of *F. occidentalis* and *M. persicae* were positive for *S. enterica*, while in the case of *M. quadrilineatus*, the contamination rate was slightly lower (47%). Hypothesis testing with *Z*-scores (*F. occidentalis Z*=0.45, *M. quadrilineatus Z*=-0.99, *M. persicae Z*=0.64) suggest that rates of contamination were not significantly different when compared to the remaining 50% of the corresponding insect population. 

**Figure 5:**
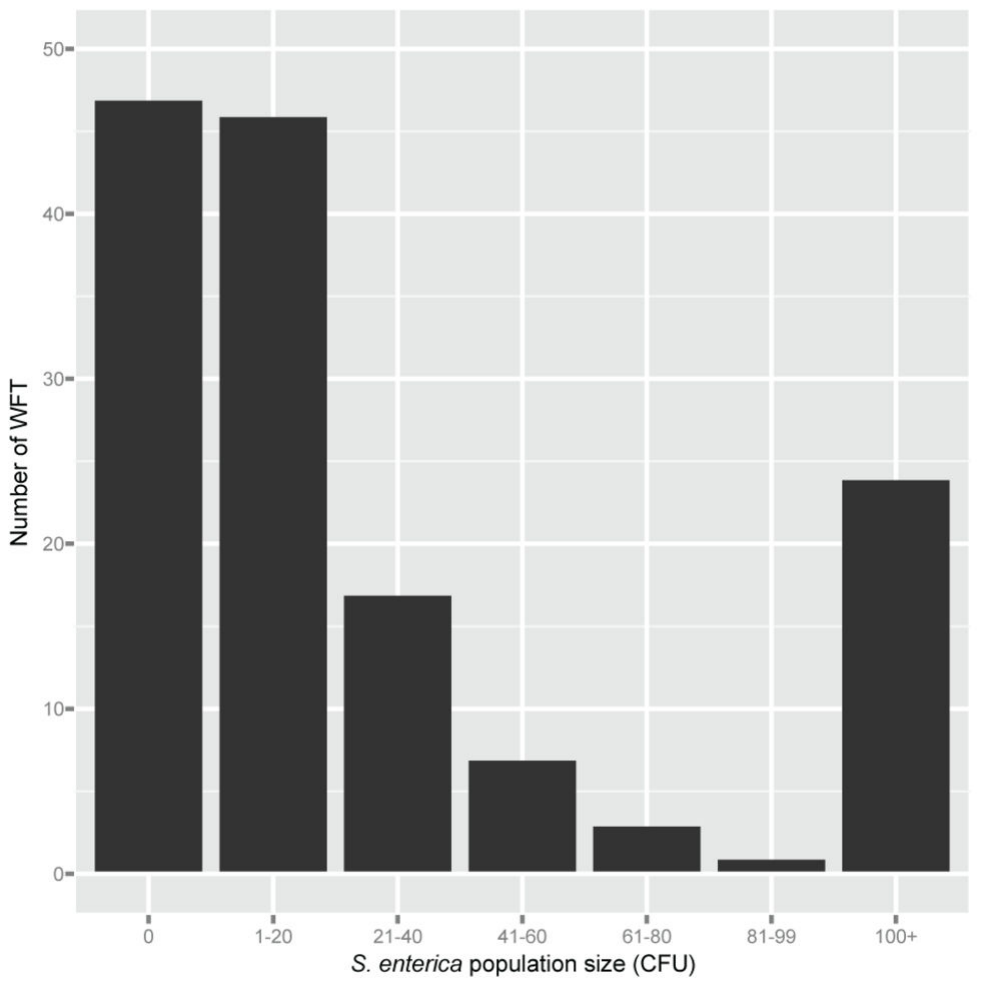
Frequency distribution of *Salmonella enterica* population size per *Frankliniella occidentalis*. Shown is the number of *F. occidentalis* (N=145) from which specific *S. enterica* populations were recovered after 24 h acquisition access period. Populations represent the number of *S. enterica* CFU counted per individual *F. occidentalis* homogenized, except for 100+ that also include populations that were too high to count. The zero column are those *F. occidentalis* which were not carrying *S. enterica* or whose populations were below the level of detection without enrichment. Data from three independent experiments were combined.

 To further characterize the potential for insect contamination with *S. enterica*, *S.* enterica populations were enumerated from individual insects following feeding on contaminated plant tissue. *F. occidentalis* were used in this experiment because they are relatively tiny and slender (usually 1-2 mm long), and represent the smallest in size of the insects evaluated in this study. *S. enterica* was not isolated from untreated control treatments. Based on direct plating, 68% (98 out of 145) of the *F. occidentalis* tested positive for contamination of *S. enterica*, while 32% (47 thrips) tested negative (Figure 5). Populations between 1-20 CFU’s were recovered from more than 30% (46 thrips) of the *F. occidentalis*; however, this value could be higher considering that insects that tested negative were not subjected to enrichment methods and could have had *S. enterica* counts below the detection limit. Interestingly, 17% (24 thrips) of the *F. occidentalis* harbored more than 10^2^ *S. enterica* CFU on their bodies (Figure 5). It is important to note that this method does not allow us to distinguish from *S. enterica* contamination of insect body versus cells ingested. 

## Discussion

In this study, *F. occidentalis*, *M. quadrilineatus*, and *M. persicae* were used as model systems to study the role of insects as potential biomultipliers of *S. enterica* on plants. Thrips, leafhoppers, and aphids were selected because they are both common agricultural pests and vectors of phytobacterial pathogens of several agricultural crops, including lettuce [20-22,34-36]. These insects have two distinct types of mouthparts and unique feeding strategies that enabled comparison of feeding behaviors and their respective influence on the colonization of plants by *S. enterica*.

 It was observed that feeding damage caused by *F. occidentalis* enhanced survival of *S. enterica* in comparison with areas of the same plant where feeding lesions were not visible. *F. occidentalis* have rasping-sucking mouthparts and feed by rasping the surface of the leaves and ingesting fluids of the mesophyll and epidermal cells of leaf tissues [37,38]. Specifically, the thrips mandible pierces a hole in the leaf and cell contents are ingested via a cibarial pump which extracts cellular contents through maxillary stylets [39]. However, the feeding process combines periods of probing or stylet penetration, and non-probing [38], and the cell damage is correlated to the frequency of probing and the duration of each probe, from the time of insertion to removal of the maxillary stylets [38]. We hypothesized that *F. occidentalis* feeding damage allowed *S. enterica* survival by providing direct access to cellular cytoplasm for successful colonization. However, it is also recognized that this feeding behavior can result in an extreme plasmolysis leaving completely empty cells, which after a short period of time, cannot provide appropriate nutrients to sustain growth of the pathogen. This may explain why similar declines in *S. enterica* population rates were observed among plants exposed to both infested and uninfested treatments. It is possible that the sampled tissue from *F. occidentalis*-infested plants was too damaged or damaged for a period of time to render the cells devoid of nutrients and thus, unable to support populations of *S. enterica*. 

 Several studies using electrical penetration graph analysis have demonstrated that thrips females feed more frequently and intensively than males, which is reflected in the production of more silvering scars [37,38]. In our study, *F. occidentalis* females were predominantly used because they are larger than males, which facilitated their handling. Hence, higher levels of insect lesions produced by females might have positively impacted *S. enterica* survival in lettuce plants in the current study. Damage to plant leaves during oviposition might have created additional wounds that served as cell entry sites for the bacteria. Therefore, it is possible that persistence of human pathogens on plants is influenced by insect size differences between the sexes and the sex-ratio of thrips populations, as well as feeding behavioral.

 It is widely recognized that pre- or post-harvest wounding and/or scarring can affect the viability, quality, and safety of fresh-cut produce. For instance, Felkey and collaborators [40] reported the inefficacy of sodium hypochlorite to eliminate contamination of *Salmonella* from stem scars and wounded tomato fruits. Interestingly, the preference of thrips for thrips-damaged leaves over fresh leaves has been previously reported as a mechanism that enables the uptake of symbiotic gut bacteria [41]. In our study, significant *S. enterica* populations were recovered from *F. occidentalis*-damaged areas of lettuce leaves. Although *F. occidentalis* lesions may appear inconsequential to consumers, our results suggest that thrips feeding scars not only reduce aesthetic quality, but can also serve as potential reservoirs of human pathogenic bacteria on plants and may increase the food safety risk. 

 Results from the current study suggest that human bacterial pathogen survival can be influenced by the presence of specific phytophagous insect taxa, with unique feeding strategies. We initially hypothesized that feeding of both *M. persicae* and *M. quadrilineatus* may fail to induce *S. enterica* persistence or growth in the phyllosphere, on the premise that they do not facilitate direct access to cellular cytoplasm for pathogen use. Most hemipterans depend exclusively on phloem sap as their primary source of nutrients, and they possess highly modified piercing-sucking mouthparts that allow them to ingest ﬂuids from plant vascular, epidermal, and/or mesophyll cells [17]. Mouthparts consist of a needle-like stylet bundle and a salivary canal that are used to ingest plant fluids and also deliver saliva into the feeding site. However, substantial differences among hemipteran feeding mechanisms have been described [17,42]. Interestingly, we found different effects on *S. enterica* persistence on leaves infested with *M. persicae* or *M. quadrilineatus*. It is possible that differences in stylet penetration behaviors could influence *S. enterica* survival in this study. Unlike the intercellular penetration style of sternorrhynchans like *M. persicae*, the intracellular style of auchenorrhynchan stylets, such as those of leafhoppers [42], could have benefited *S. enterica* through leaking of phloem sap, similar to that which can occur with feeding by *F. occidentalis*. 

 Feeding behavior, instead of mouthpart type, may correlate with human bacterial pathogen survival in infested leaves. Miles [43] described two different feeding strategies used by all hemipterans: “sheath feeding” in which insects protect their stylet tips with a sheath made of solidifying saliva, and “lacerate-and-flush” feeding in which stylets puncture plant tissues and rupture cellular matter, while releasing watery saliva, and then ingest the resulting fluid. Later, Backus et al. [42] renamed the second strategy as cell rupture feeding. Although, it has long been thought that the sheath feeding is the primary strategy used by most auchenorrhynchan species, various studies have more recently reported *Empoasca* spp. leafhoppers (Hemiptera: Cicadellidae), as cell rupture feeders, not salivary sheath feeders [42,44]. This feeding strategy involves ingestion of mesophyll cell contents, and comprises two sub-strategies that vary in duration and intensity of cell laceration, which can be alternated on different tissues or host plants [42]. Although it has not yet been described, it is possible that in our study *M. quadrilineatus* used an intermediate feeding strategy that allowed the enhanced persistence of *S. enterica* by causing less drastic mesophyll cell damage and release of phloem-sap contents. Additionally, it is possible that the larger stylets of *M. quadrilineatus*, compared to *M. persicae*, caused more physical damage to plant tissues and introduced more *S. enterica* cells into damaged tissues, where the pathogen had access to nutrients.

 Plant defense response to herbivores may influence human pathogen populations. Feeding strategies that cause more aggressive damage to plant tissue, such as chewing, rasping-sucking, or repeated perforation of multiple plant cells stimulate the plant’s jasmonic acid dependent and -independent wound-responses [17,45]. Several types of Lepidopteran caterpillars, Coleoptera, Tetranychid mites, Thysanoptera, and certain other Hemipteran leafhoppers are known to cause these types of injuries when feeding on plant material [17,46,47]. On the other hand, other Hemipteran insects such as whiteflies and some aphids follow intercellular pathways in the leaf as they probe for suitable feeding sites, causing minimal to unnoticeable cellular damage [17]. This type of feeding behavior mimics infection processes of biotrophic phytopathogens and usually plants respond with salicylic acid (SA) dependent pathways [17,46,48]. It is possible that plant-wounding responses induced by *M. quadrilineatus* feeding could have indirectly benefited *S. enterica* colonization of lettuce plants by antagonizing defense responses associated with pathogen establishment and infection, such as SA-dependent and –independent defenses and pathogenesis related proteins. Plant response to thrips and aphid feeding involves several signaling pathways associated with both pathogen infection and wounding [45,46]. Moreover, Mouttet et al. [47] reported that production of secondary metabolites by rose (*Rosa hybrida* cv. Sonia) plants previously infected with a plant pathogen could have had an adverse effect on aphids and thrips feeding. However, Erickson and collaborators observed lower *E. coli* O157:H7 populations internalized within leaves previously exposed to insects, including aphids and thrips [18]. In our study, *S. enterica* populations, among plants exposed to *F. occidentalis* or *M. persicae*, were similar to those without insects, suggesting that defense responses induced by aphids or thrips do not have a relevant effect on *S. enterica* populations. Moreover, whether *S. enterica* contamination of plants positively or negatively affects the behavior of phytophagous insects remains unknown.

 It seems likely that only insect feeding behaviors that cause direct damage to plant cells tended to enhance the longevity of *S. enterica* on lettuce. However, the ability of phytophagous insects to become contaminated with the human pathogen seems to be independent of feeding strategy used. It was demonstrated that *F. occidentalis*, *M. quadrilineatus*, and *M. persicae* could become contaminated with *S. enterica* from contaminated plant tissues including lettuce leaf and green bean pods. Particularly, *F. occidentalis*, the smallest of the insects tested, harbored large *S. enterica* populations after a 24 h access period to contaminated plant material. It is well know that adult thrips are not strong flyers; however, they are quite active and can move quickly on the surface of leaves. In fact, the wandering behavior, which involves roaming, scraping of their heads, and search for new-feeding sites, is characteristic of thrips when they are not probing [37]. This, in addition to their thigmotactic behavior that brings them in close contact with their host plant [49], suggests their potential to influence the persistence and potentially the spread of *S. enterica*-adhered to their bodies over leaf surfaces and flowers. It is acknowledged that the use of high inoculum concentrations that are unlikely to occur in natural environments could have increased the probability of movement of bacteria by insects. Nevertheless, it is important to emphasize that bacterial populations at the time of infestations were similar to concentrations of *Salmonella* recovered from drainage water [50] and wound-inoculated tomatoes after treatment with chlorine water [40]. Taken together, these results highlight the potential role of insect pests of agricultural crops to influence the population dynamics of the human pathogen, *S. enterica*. Although in this study it was not determined whether *S. enterica* could adhere to the outside of phytophagous insects or be ingested, the potential for these insects to be biological vectors of *S. enterica* remains to be determined. Furthermore, since *S. enterica* was recovered from insect bodies and insect damaged plant material, insects or insect-damaged plant tissue could be exploited as a novel sentinel strategy for *S. enterica*-contaminated crop monitoring.
